# Book Reviews

**DOI:** 10.1155/S1463924678000140

**Published:** 1978


### Book Reviews

### Quality Control in the Clinical Laboratory; A Procedural Text

**by Paul J. Ottaviano and Arthur F. Disalvo**

*1978, University Park Press, Baltimore, London, Tokyo. Pp 223, $14.95, ISBN 0-8391-1156-8.*

**This** is a book of value to all clinical laboratory workers, but its title could be misleading for those outside the USA. It infers that procedures are given which have general worldwide application, while the authors must have had in mind solely readers in the USA; a more appropriate title might be "Compliance with US Clinical Laboratory Quality Legislation". However, the US approach is sound and there is much in it to be commended for countries which have not proceeded so far.

Authority is considerably enhanced by quotations from, and the obvious involvement of, many members of the staff of the Center for Disease Control (CDC), Atlanta. The book gathers together a good selection of commercial, official and semi-official tables, listings and charts; in fact, 106 out of a total of 223 pages are taken up by an appendix containing the CDC check list for clinical laboratory certification, together with the appropriate Federal regulation which requires compliance with each item. A further involvement of the CDC is the inclusion of a daily questionnaire to aid quality control in the clinical chemistry laboratory, designed by David Bayse (presumably Boyze is a misprint), Director, Clinical Chemistry Division, CDC.

In general, quality control systems are given as used in the authors' laboratory, and coverage includes blood bank, clinical chemistry, haematology, medical microbiology, immunology and urinalysis. The subject is dealt with in a logical sequence. Firstly, attention is drawn to the need for quality control by quoting statistics for poor laboratory performance found in a survey completed as recently as 1976. Then considered in turn are:— control of incoming materials, equipment maintenance, statistical basis, specific requirements for individual types of laboratories and finally remedial action. There is a refreshing concentration upon preventative and management aspects. It is important to note that with the Appendix, the book only contains 105 pages of text and covering as it endeavours to do, most branches of clinical laboratory work, an in-depth approach is impossible. The preface states that the purpose is to provide a step-by-step procedure for establishing a programme that will meet the minimum requirements for satisfactory quality control as currently required in the USA. In doing just that, much of the underlying philosophy is not given and many important items, for example the types and value of various charts, including cusum, are not discussed.

Under clinical chemistry there is no mention of the new and increasingly important reference technology, or the IFCC recommendations on methods of assessing the quality of a method are not dealt with. The bibliography contains only 32 items, mostly standard works and documents, and the wealth of published material which has appeared in international journals over the last decade, is scarcely mentioned.

It is difficult to agree with the statement in the synopsis on the fly-leaf that the book is highly recommended as a text for advanced studies in medical technology. A much more comprehensive approach would be required to merit such a claim, but nevertheless, any laboratory which follows the protocols given will certainly have instituted good and sound coverage. Text books on clinical laboratory quality control are rare and this one undoubtedly has a place.

*F. L. Mitchell*

### Computing in Clinical Laboratories

**Edited by F. Siemaszko**

*1978, Pitman Medical, Tunbridge Wells, Kent. Pp 302, £10.00. ISBN 0-272-79502X*

**This** book is the report of the proceedings of the Second International Conference on Computing in Clinical Laboratories which was held in Birmingham in September 1977. The book is divided into seven sections each dealing with various aspects of Clinical Laboratory Computing. Thirty three different presentations are made and reflect the experience of different contributors in the medical, electronic, engineering, chemical, micro-biological and statistical fields.

With so many contributors it is impossible within this review to comment on each presentation, but since the organisers of the conference laid down broad categories then comments will be made within those classifications. The first section, dealing with system design and implementation forms a very useful introduction and background against which new or potential users can assess their own computing requirements. Under the section on microprocessors the reader is introduced in some detail to this new computing tool. A number of applications and potential applications are described. Undoubtedly these processors are going to play an increasing part in laboratory automation and it is important that laboratory directors should be aware of their possibilities.

The next section on "cost effectiveness" deals with the problem of evaluation of computer systems. This section is of a general nature dealing more with the aims and philosophy of evaluation and outlining some of the temporal difficulties associated with such work. A report on SMAC cost effectiveness, whilst interesting, is somewhat out of place since this instrument is predominantly a chemical analyser. The section on input/ouput devices should be of great value to potential readers, pointing quite clearly the direction in which such systems are likely to go in the near future.

Problems relating to interfacing and remote processing are dealt with briefly and supported by examples. The section on the choice and use of laboratory results provides an insight into the future capabilities of laboratory computers. Unfortunately within this section contributors have used a large number of abbreviations and have provided references which require to be taken up before sense can be made of some of the articles. Irrespective of this criticism this section makes interesting and stimulating reading. The last section deals with recent advances in numeric techniques and is an area which is beginning to play more and more part in the evaluation and correct calculation of results.

With contributors from so many different disciplines it is inevitable that this book will have something of interest to a wide range of readers. It will be required reading for most laboratory directors, either those starting in the field of laboratory computing or those already deeply engaged in this activity. Inevitably in such a complex and technical field a small number of typographical and other errors are evident. These do not have any significant effect upon the interpretation of the typescript.

This book deserves to be, and should be, found on the shelves of most departmental libraries.

*L. B. Roberts*

